# Supplementary material for: Modelling Skylarks (Alauda arvensis) to Predict Impacts of Changes in Land Management and Policy: Development and Testing of an Agent-Based Model
Source: PLoS One. 2013 Jun 6;8(6):e65803. doi: 10.1371/journal.pone.0065803 (PMC3675089; doi:10.1371/journal.pone.0065803)
Supplement: Supporting Information S4 — The skylark ODdox as a zipped archive. (ZIP) [file pone.0065803.s004.zip › Skylark_ODdox/plants_8cpp.html]

ALMaSS Skylark ODdox: plants.cpp File Reference


ALMaSS Skylark ODdox
 2.0


- Main Page
- Related Pages
- Classes
- Files

- File List
- File Members

Macros |
Functions |
Variables

plants.cpp File Reference

`#include <cstdio>`  
`#include <math.h>`  
`#include <stdlib.h>`  
`#include "configurator.h"`  
`#include "maperrormsg.h"`  
`#include "plants.h"`  
`#include "tov_declaration.h"`

|  |  |
| --- | --- |
| Macros | |
| #define | \_CRT\_SECURE\_NO\_DEPRECATE |

|  |  |
| --- | --- |
| Functions | |
| void | FloatToDouble (double &, float) |

|  |  |
| --- | --- |
| Variables | |
| class CropData \* | g\_crops |

---

## Macro Definition Documentation

#define \_CRT\_SECURE\_NO\_DEPRECATE

---

## Function Documentation

|  |  |  |  |
| --- | --- | --- | --- |
| void FloatToDouble | ( | double & | , |
|  |  | float |  |
|  | ) |  |  |

{

char \* num = 0;

num = new char[\_CVTBUFSIZE];

errno\_t err = \_gcvt\_s(num, \_CVTBUFSIZE,f,8);

if (err!=0) {

assert(0);

}

d = atof(num);

delete [] num;

}

---

## Variable Documentation

class CropData\* g\_crops

Referenced by BeetleBank::BeetleBank(), VegElement::DoDevelopment(), UnsprayedFieldMargin::DoDevelopment(), FieldBoundary::FieldBoundary(), VegElement::ForceGrowthInitialize(), VegElement::ForceGrowthSpringTest(), CropRotation::GetFirstCrop(), Heath::Heath(), HedgeBank::HedgeBank(), Hedges::Hedges(), Landscape::Landscape(), Marsh::Marsh(), NaturalGrass::NaturalGrass(), Orchard::Orchard(), OrchardBand::OrchardBand(), OrchardGrass::OrchardGrass(), PermanentSetaside::PermanentSetaside(), VegElement::SetGrowthPhase(), VegElement::SetVegType(), UnsprayedFieldMargin::UnsprayedFieldMargin(), VegElement::VegElement(), YoungForest::YoungForest(), and Landscape::~Landscape().


- CJT
- MSVC
- ALMaSS Working Source
- Landscape
- plants.cpp
- Generated on Thu Jan 10 2013 13:15:35 for ALMaSS Skylark ODdox by
   1.8.1.1
